# Highlight: Cracking the Shell of the Mysterious Argonaut Octopus

**DOI:** 10.1093/gbe/evac156

**Published:** 2022-11-04

**Authors:** Casey McGrath

**Fig. 1.**
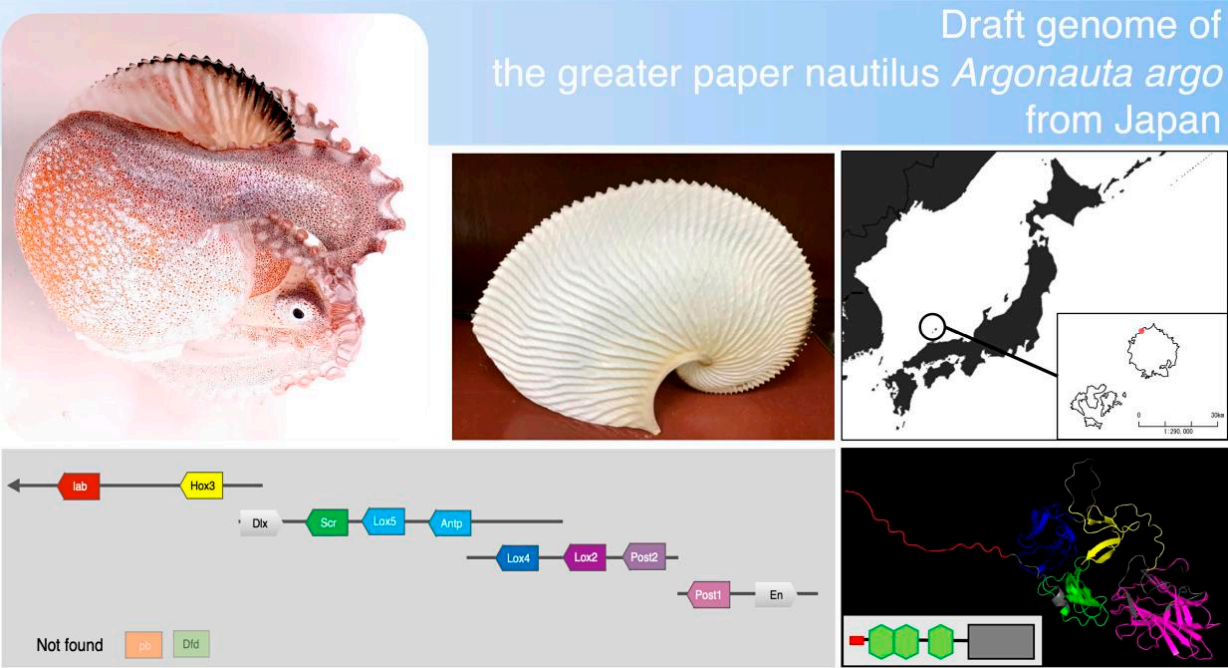
The draft genome of the greater argonaut or paper nautilus, *Argonauta argo*, revealed several surprises. Shown are photographs of *A. argo* and its paper-thin eggcase; a map of the location off Oki Island, Japan, where the sequenced specimen was caught; the nearly intact Hox gene cluster, which was previously thought to be fragmented in all octopuses; and a depiction of the *A. argo* LamininG3 protein, which is homologous to a protein in the nautilus shell but does not appear to be present in the eggcase of *A. argo*.

The greater argonaut, *Argonauta argo*, has a reputation for being the world's weirdest octopus and indeed may be one of the most unusual and mysterious creatures to roam the ocean. Unlike its relatives that live on the seafloor, this octopus has a pelagic lifestyle, meaning that it lives in the open ocean. This transition led to a host of evolutionary adaptations that make the argonaut unique among its octopod relatives. The most obvious of these is the presence of a paper-thin, spiral-shaped structure secreted by argonaut females which is similar in appearance to the spiral shell of the nautilus (fig. 1). This shell-like “eggcase” protects the eggs laid inside and takes in air to maintain buoyancy in the water column; it has also given rise to a common misnomer for the argonaut: the paper nautilus. Weirder still, in order to mate, an argonaut male detaches his reproductive arm, known as the hectocotylus, and gives it to the female, who keeps it inside her shell and uses it to fertilize her eggs. While scientists have long been interested in learning more about these unusual invertebrates, there have been relatively few studies on argonauts due to the difficulty in maintaining them in aquaria and obtaining wild-caught samples. Now, in a new study in *Genome Biology and Evolution*, researchers from six Japanese institutions present the first draft genome of *A. argo*, providing new insight into the evolution of its unusual characteristics while deepening our understanding of cephalopods (octopuses, cuttlefish, squid, and nautiluses) and mollusks (Yoshida et al. 2022).

Led by corresponding authors Masa-aki Yoshida from Shimane University and Davin Setiamarga from National Institute of Technology, Wakayama College, the researchers set out to better understand the evolution of the argonaut, particularly the provenance of its shell-like eggcase. The argonaut eggcase differs from the shell of the nautilus in its structure, composition, and method of formation. For example, rather than being secreted from the mantle (body) like the nautilus's shell, eggcase proteins are secreted from two of the argonaut's arms. Due to these differences, researchers had long suspected that the argonaut eggcase had evolved independently from the shell of the nautilus. According to Setiamarga, “it was not yet known how such a complex morphological trait was reacquired during evolution, particularly at the molecular level.” Luckily, the team was well-positioned to conduct a genomic study on argonauts. “Argonaut samples can be collected as bycatch from fishing nets on the shores of Oki Island in Shimane Prefecture, Japan, where one of the major authors (Yoshida) is located. Having access to fresh argonaut samples has made it possible for us to study this unusual organism.”

Analysis of the new draft genome of *A. argo* produced a number of surprising results. First, the *A. argo* genome was less than half the size of other cephalopod genomes, making it the smallest cephalopod genome known to date. Other cephalopods that have been sequenced so far exhibit relatively large genomes containing a high proportion of repetitive elements (∼45% in *Octopus bimaculoides*). As also noted by Yoshida, “Our discovery of a species with a smaller genome size in the octopus lineage indicates that this trend [of genome expansion] is not essential for cephalopod evolution.” The authors found additional differences between the *A. argo* and *O. bimaculoides* genomes in their arrangements of Hox genes—genes critical in development whose sequence and order tend to be highly conserved—further suggesting that there is greater variation in genomic architecture among octopods than previously believed.

The authors also found highly conserved gene clusters of reflectins and tyrosinases, two gene families that they argue are associated with the argonaut's transition to a pelagic lifestyle. Living in the open ocean, the argonaut relies heavily on its camouflage abilities to avoid predators. In particular, one of the argonaut's arms usually wraps around its shell and reflects light using iridescent chromatophores, causing it to look like a mirror; the reflectin gene cluster likely plays a key role in this process. The tyrosinases identified by the authors are related to proteins that can be found in the shell matrix of some mollusks. Although it remains to be confirmed that these genes are involved in eggcase formation in the argonauts, the authors hypothesize that these genes were conserved in the argonaut's non-shell-producing octopod ancestor and then repurposed for the argonaut's shell-like eggcase.

The study's authors also compared the evolutionary history of genes encoding eggcase matrix proteins in *A. argo* and those encoding shell matrix proteins in the nautilus and other mollusks. In general, eggcase matrix proteins were homologous (descended from a common ancestor) to genes present in other cephalopods and mollusks, though they were not used in shell formation in these species. Similarly, homologs of shell matrix proteins were present in *A. argo*, although they were not involved in eggcase production. These results “support the previously suggested hypothesis that the argonaut octopods recruited proteins unrelated to shell formation and used them for their eggcase,” according to Kazuki Hirota, a graduate student at The University of Tokyo who was also involved in the study.

The authors anticipate that their study will pave the way for future studies in several previously unexplored areas, including argonaut epigenomic regulation (changes in gene expression independent of changes in the DNA sequence), functional studies of shell and eggcase-forming genes, and ultimately analyses of the population dynamics of *A. argo*, a cosmopolitan species that is widely distributed throughout the Mediterranean, Australia, and the western Pacific. In addition, there is considerable interest in the sexual dimorphism of the argonauts given their unusual mode of reproduction. Unfortunately, such studies are likely to be hindered by the inability to raise and breed argonauts in captivity and the lack of accessibility to wild-caught samples. Nonetheless, as noted by Yoshida and Setiamarga, “There are a lot of intriguing questions to be addressed. We anticipate that the availability of the genome data of the argonauts will help us to understand not only this species, but also the cephalopods and mollusks in general.”
